# Exercise training program in children and adolescents with asthma: a randomized clinical trial

**DOI:** 10.1590/1984-0462/2026/44/2026014

**Published:** 2026-11-09

**Authors:** Mariana Mazzuca Reimberg, Rebeca Souza Scalco, Bruno Ramos Salu, Andrey Jorge Serra, Gustavo Falbo Wandalsen, Dirceu Solé, Simone Dal Corso, Fernanda de Cordoba Lanza

**Affiliations:** a Universidade Nove de Julho, São Paulo, SP, Brazil.; b Universidade Federal de São Paulo, São Paulo, SP, Brazil.; c Monash University, Melbourne, Australia.; d Universidade Federal de Minas Gerais, Belo Horizonte, MG, Brazil.

**Keywords:** Asthma, Pediatric, Functional capacity, Physical exercise, Quality of life, Pulmonary rehabilitation, Asma, Pediatria, Capacidade funcional, Exercício físico, Qualidade de vida, Reabilitação pulmonar

## Abstract

**Objective:**

To evaluate, in children and adolescents with asthma, the effects of an 8-week exercise training program on exercise capacity.

**Methods:**

Single-blind, randomized clinical trial in participants with asthma between six and 17 years old under regular treatment. The intervention group performed supervised aerobic and resistance training, and the control group performed supervised stretching in 60-minute sessions twice a week for eight weeks. They were assessed at baseline and following the intervention. The primary outcomes were (VO_2_peak) and distance walked on the Modified Shuttle Test (MST). Secondary outcomes included quality of life, physical activity in daily living, and serum inflammatory profile. The analyses were intention-to-treat and per protocol.

**Results:**

Thirty participants in the intervention group and 29 in the control group, mean age 10±3 years old, normal lung function (forced expiratory volume — FEV_1_/forced vital capacity — FVC: 86±8), Global Initiative for Asthma (GINA) step: 2 (1–3). No significant differences between groups were observed at the end of the protocol regarding all outcomes. The VO_2_ peak was 40.3±12.4 mL/kg in the intervention group and 39.2±16.0 mL/kg in the control group (p=0.764). The distance walked on the MST was 852±219 m for the intervention group and 759±255 m for the control group (p=0.142).

**Conclusions:**

A twice-weekly, 8-week exercise program for children and adolescents with asthma did not improve exercise capacity, quality of life, or the inflammatory profile. The optimal frequency of intervention for this population requires further investigation.

## RESUMO

**Objetivo:**

Avaliar, em crianças e adolescentes com asma, os efeitos de um programa de treinamento físico de oito semanas na capacidade de exercício.

**Métodos:**

Ensaio clínico randomizado, simples-cego, com participantes com asma de 6 a 17 anos de idade. O grupo de intervenção realizou treinamento aeróbico e resistido e o grupo controle realizou alongamento, em sessões de 60 minutos, duas vezes por semana, durante oito semanas. Os desfechos primários foram o consumo de oxigênio de pico (VO_2_pico) e a distância percorrida no Shuttle Test Modificado (STM). Desfechos secundários: qualidade de vida, atividade física na vida diária e perfil inflamatório sérico. As análises foram realizadas por intenção de tratar e per protocol.

**Resultados:**

Trinta participantes no grupo intervenção e 29 no grupo controle, média de idade de 10±3 anos, função pulmonar normal (volume expiratório forçado — VEF_1_/capacidade vital forçada — CVF: 86±8), etapa da *Global Initiative for Asthma* (GINA): 2 (1–3). Não foram observadas diferenças significativas entre os grupos ao final do protocolo com relação a todos os desfechos. O VO_2_ pico foi de 40,3±12,4 mL/kg no grupo intervenção e de 39,2±16,0 mL/kg no grupo controle (p=0,764). A distância percorrida no STM foi de 852±219 m para o grupo intervenção e de 759±255 m para o grupo controle (p=0,142).

**Conclusões:**

Um programa de exercícios realizado duas vezes por semana, durante oito semanas, não melhorou a capacidade de exercício de crianças e adolescentes asmáticos, nem a qualidade de vida ou o perfil inflamatório.

## INTRODUCTION

Asthma is a chronic lung disease that can occur in childhood. Globally, approximately 14% of children and adolescents experience asthma symptoms, but there are high-prevalence areas reaching 20–25% (Latin and North America).^1^ It is characterized by airway inflammation,which is associated with reduced functional capacity, impaired quality of life, and sedentary behavior.^2,3,4^

In a systematic review of patients with asthma undertaking physical training lasting for 20–30 min, 2–3 times a week, for 6–16 weeks, the training was shown to improve cardiopulmonary fitness and quality of life without adverse effects.^5^ However, this systematic review showed some inconsistent findings regarding improvements in exercise and functional capacity.^6,7,8,9,10,11,12^

The cardiopulmonary exercise test (CPET) is the gold standard to assess exercise capacity used in many studies, and one alternative to assess functional capacity is the modified shuttle test (MST). Although these tests are established for asthmatic patients,^3,6^ information is lacking regarding its use after exercise programs in pediatric populations.

Beyond exercise capacity, physical activity significantly influences the inflammatory profile of pediatric patients.^13^ For instance, children with asthma showed reduced levels of superoxide dismutase and total plasma nitric oxide following an eight-week exercise program.^14^ In contrast, studies by Andrade et al.^15^ and Moreira et al.^16^ detected no significant changes in serum interleukins after aerobic training in adolescents with asthma. Due to these conflicting results, a gap remains in determining whether exercise training effectively modulates the systemic inflammatory profile in this population.

Despite existing evidence, the chronic effects of exercise, incorporating multidimensional physiological parameters, have yet to be simultaneously evaluated in pediatric asthma patients. Therefore, the objective of this study was to investigate whether, in children and adolescents with asthma receiving regular pharmacological treatment, an exercise training program would improve exercise and functional capacity, quality of life, physical activity in daily life, and the serum inflammatory profile after eight weeks of intervention.

## METHOD

This study was a parallel-group, single-blind, randomized controlled trial conducted from March 2015 to November 2018 at the Pulmonary Rehabilitation Laboratory at Universidade Nove de Julho (UNINOVE), São Paulo, Brazil. The trial was prospectively registered at ClinicalTrials.gov (NTC02383069).

Although the submission process began in February 2015, official registration was granted in March 2015. Patients were enrolled starting March 9, 2015. The Consolidated Standards of Reporting Trials (CONSORT) guidelines checklist was followed for this trial report.

Participants were between six and 18 years old and were recruited from the Allergy, Clinical Immunology, and Rheumatology Clinic at the Pediatric Department of UNIFESP, São Paulo, Brazil. Inclusion criteria were a physician-confirmed diagnosis of asthma (GINA, Steps 1–5),^1^ regular treatment for at least six months prior to the study, and consistent medication adherence for at least the previous three months. Exclusion criteria were failure to complete the protocol evaluations; interruption of medical care or pharmacological treatment; presence of acute lung infections, other chronic lung diseases, or comorbidities (e.g., neuropathies or heart disease); and engaging in exercise more than three days per week.

Participants were enrolled in the study after their legal guardians read, agreed to, and signed the informed consent form. The study was approved by the Institutional Review Board (protocol No. 738192/2014, Certificate of Presentation for Ethical Appreciation — CAAE: 31907614.4.0000.5511).

The primary outcomes were physical exercise capacity, assessed by cardiopulmonary exercise testing (VO_2peak_ and peak workload), and distance walked on the MST. The secondary outcomes were quality of life, serum inflammatory profile (IL-4, IL-5, IL-10, IL-13, and TNF-^®^), and physical activity in daily life (sedentary time and level of physical activity).

Assessments were conducted over three visits. On Day 1, spirometry, the CPET, a quality of life and an asthma control questionnaire were applied. Day 2 occurred 48 hours after Day 1 and included spirometry (to guarantee that the baseline clinical status remained stable), the MST, and accelerometer placement. On Day 3, seven days after Day 2, the accelerometers were returned, and the inflammatory profile was assessed. Participants were evaluated at baseline and after the eight-week intervention. The intervention assessments started within one week after the end of the intervention.

The pediatric asthma quality of life questionnaire (PAQLq) is composed of 23 questions divided into three domains: physical activity limitations (five questions), symptoms (ten questions), and emotions (eight questions). The total score was used for analysis; it is a 7-point scale, with 1 indicating the maximum loss and 7 indicating no loss in quality of life.^17^

The Asthma Control Test (ACT or C-ACT)^18,19^ consists of five and seven questions, respectively, with scores for each item ranging from 1 to 5 points. According to the questionnaire, asthma was considered controlled if the score was greater than 20, partially controlled if the score was between 16 and 19, and uncontrolled if the score was 15 or lower.

The spirometry test was performed according to recommendations^20^ using Ultima CPX equipment (MedGraphics Corporation^®^, St. Paul, MN, USA). All participants performed the maneuvers pre- and post-bronchodilation (salbutamol 400 μcg). Forced vital capacity (FVC), forced expiratory volume at the first second (FEV_1_), FEV_1_/FVC ratio, and forced expiratory flow (FEF_25-75_) in absolute and percentage of the predicted values^21^ were assessed.

The CPET test was performed on an electromagnetic braking cycle ergometer (Corival^®^, LODE BV Medical Technology, Groningen, Netherlands) connected to gas exchange analysis according to recommendations^22^ (Breeze CardiO2 System^®^ microcomputer, Medical Graphics Corporation-MGC, St. Paul, MN, USA). After 2 minutes of freewheel load, the load was increased (5 to 20 watts/minute) according to participant characteristics, and the test was ended with maximal exhaustion, which would be between 8 and 12 minutes. A maximal test was considered valid if the peak respiratory exchange ratio (VCO_2_/VO_2_) was greater than 1.0 for children and 1.1 for adolescents, the peak heart rate (HR) was higher than 90% of the predicted value, and the elevated rating of perceived exertion was reached. The oxygen uptake (VO_2_, mL min^-1^) was continuously recorded, as well as the workload at the end of the test.

The MST was performed to determine the functional capacity. It was assessed in a 10-meter-long corridor, as described.^23^ It is an incremental test, externally paced by an audio signal, in which the participant could walk or run. The test was performed twice on the same day, with a 30-minute interval (learning effect). HR and SpO_2_ were continuously evaluated. The distance walked, in absolute and percentage of the predicted value,^24^ was recorded. Normal functional capacity was defined as a distance walked ≥80% of the predicted values.

The participants were monitored using an accelerometer (ActiGraph, GT3X, FL, USA) over seven consecutive days to assess physical activity in daily living. It was considered as successfully monitored when they used the accelerometer for at least five days for more than 8 hours/day in a waistband.^25^ The collected data were slow and light physical activity, moderate to vigorous physical activity, and the total number of steps. Sedentary behavior was considered <8,000 steps/day.^25^

Interleukins (IL) 4, 5, 10, 13, and 17, as well as tumor necrosis factor (TNF)-α were analyzed in a blood sample using MILLIPLEX^®^MAP (#MMHMAG-44K, Merck Millipore Corporation, Darmstadt, Germany). The data were interpreted using xPONENT^®^ software (Luminex, IL, USA), and the samples were expressed as pg/mL.

The participants were randomly allocated to an intervention or control group using a computer-generated random number. A researcher who was not involved in the trial concealed the random allocation sequence in sequentially numbered, opaque, sealed envelopes. After the baseline evaluation, the physiotherapist responsible for delivering the protocol opened the sealed envelopes and assigned the participants to the intervention or control group. Neither the outcome assessor nor the physiotherapist who conducted the protocol was blinded.

In the intervention group, participants underwent a supervised exercise program twice a week for eight weeks (16 sessions). Each session consisted of aerobic training, resistance training, and a high-frequency oscillation device (Flutter^®^), three sets of ten repetitions. The Flutter^®^ device was chosen as the intervention for the control group to ensure a non-exercise-based approach. This allowed us to provide an active intervention that avoids bias associated with structured aerobic exercise or physical training. The target intensity for aerobic session was 60 to 80% of the maximum workload of the CEPT or of the HR reserve registered by the modified shuttle test, followed by the modified Borg scale (between 4 and 6). The exercise program could be on a treadmill or a bike, or on the floor (jumping, running, playing ball in a circuit). Each session lasted for 60 minutes, with a minimum interval of 24 hours between them.

In the control group, the participants underwent supervised stretching exercises and a high-frequency oscillation device (Flutter^®^) twice a week for eight weeks (16 sessions). Each session lasted 60 minutes, with a minimum interval of 24 hours between them.

The detailed protocol has been previously described in the published protocol study.^26^ The sample size was determined based on the run shuttle walk test levels and on the maximum load achieved in CPET as described in the published protocol.^26^

The Shapiro-Wilk test was used to determine the distribution (normal or non-normal) of the data. Primary and secondary outcomes were compared to baseline in each group and between groups at the end of treatment. Analysis was performed as intention-to-treat and *per protocol*. The variables were summarized using mean (SD). The between-group and within-group changes were expressed as mean (95%CI). Repeated-measures analysis of variance (ANOVA) with a Bonferroni *post hoc* test was used for parametric data. The participants were classified as responders if they scored above the minimal clinically important difference (MCID) for distance walked at MST (>47 m) or for workload at CPET (>10 W).^2^ The chi-square test was performed to compare volunteers who walked more than 80% of the predicted distance at MST, or more than 47 m,^23,24^ between both groups. The Statistical Package for the Social Sciences (IBM SPSS Statistics for Windows, version 20.0, NY, USA) was used in this study. A p<0.05 was considered statistically significant.

## RESULTS

Of the 272 volunteers screened, 76 were randomized (Figure 1). Regarding asthma severity, most participants in both the control (n=29) and intervention (n=30) groups were classified as GINA steps 1–3. Baseline characteristics were similar between groups (Table 1). No participants were excluded for exercising more than three times per week. No participants developed exercise-induced bronchospasm during the intervention program or assessments.

**Figure 1:**
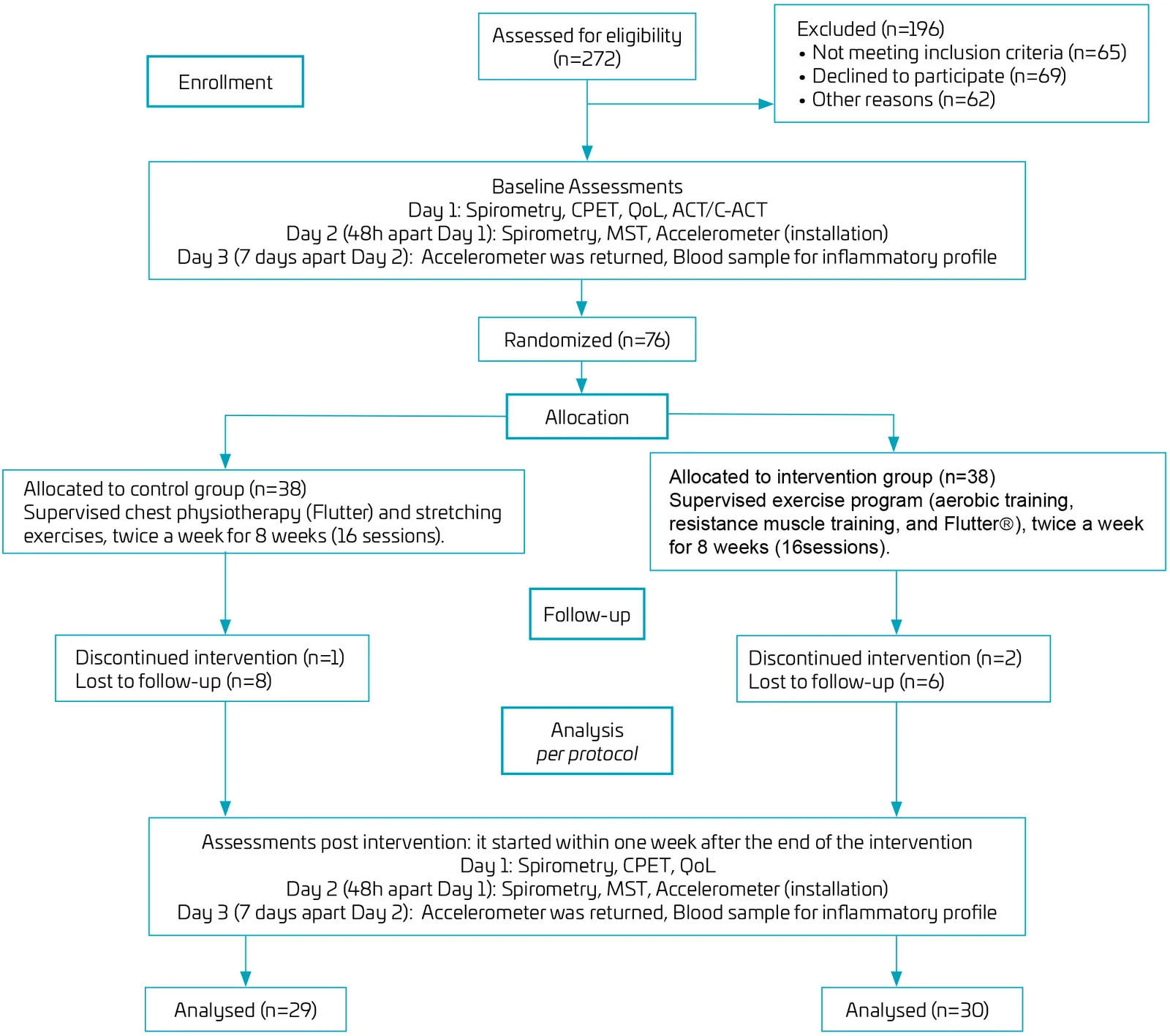
Design and flow of participants through the trial.

Source: Author’s development.

CPET: cardiopulmonary exercise test; QoL: quality of life questionnaires; MST: Modified Shuttle Test; ACT/C-ACT: asthma (children) control test.

**Table 1:** Baseline characteristics of intervention and control groups of children and adolescents who completed or did not complete the 8-week exercise training program.

Variables	Intervention group			Control group		
	Total n=38	Completed program n=30	Non-completed n=8	Total n=38	Completed program n=29	Noncompleted n=9
Age, years old	11±3	11±3	12±3	10±3	11±3	9±3
Sex (Male, n/%)	26/68	22/73	4/50	20/53	16/55	2/22
Weight, kg	46±18	45±20	48±9*	41±13	42±14	37±9
Height, cm	145±17	143±18	150±6*	142±16	144±15	133±13
BMI, kg/m2	20 (17–24)	21 (18–24)	21 (19–25)	19 (17–22)	19 (17–21)	21 (18–22)
FVC, L (% pred)	2.6±1.0 (101.1±13.5)	2.6±0.9 (101.4±13.5)	2.8±0.4* (100.3±14.3)	2.5±1.0 (99.5±12.2)	2.6±0.9 (99.0±12.4)	2.1±0.6 (101.1±12.7)
FEV_1_, L (% pred)	2.2±1.0 (94.1±14.2)	2.2±0.8 (94.0±13.0)	2.5±0.5* (94.5±18.8)	2.1±1.0 (92.4±12.4)	2.2±0.7 (92.6±11.9)	1.8±0.5 (91.9±14.9)
FEV_1_/FVC	86.0±7.3	85.9±7.3	86.5±8.0	86.4±8.0	87.0±8.6	84.6±6.6
FEF_25-75%_, L(% pred)	2.7±1.1 (96.9±30.4)	2.6±1.0 (94.5±28.3)	3.2±1.1* (104.7±37.4)	2.6±1.0 (94.5±27.2)	2.9±1.0 (98.6±28.5)	1.9±0.6 (82.4±20.1)^†^
GINA, step	2 (1–3)	1 (2–3)	2 (1–3)	2 (1–3)	2 (1–3)	2 (1–3)
GINA step 1-2, n	16	15	4	12	8	4
GINA step 3, n	10	7	3	13	11	2
GINA step 4-5, n	12	8	4	13	10	3
ACT, >12 y	20 (18–23)	19 (13–21)	18.5 (13–23)	20 (17–23)	21.5 (17–23.7)	18.5 (17–20)
C-ACT, <12 y	21 (17–23)	20 (18–22)	21 (19–25)	20 (17–22)	20 (18–22)	19 (12–22)
Inhaled corticosteroids, μcg	450 (400–800)	400 (400–00)	800 (500–1100)^‡^	450 (362–800)	400 (400–800)	650 (237–1050)

BMI: body-mass index; FVC: forced vital capacity; FEV_1_: forced expiratory volume at the first second, FEV_1_/FVC: FEV_1_/FVC ratio, FEF_25-75%_: forced expiratory flow at 25–75% of FVC curve; GINA: Global Initiative for Asthma; ACT: asthma control questionnaire; C-ACT: children asthma control questionnaire.

*vs. non-completed program of control group;

^†^vs. completed program of control group;

^‡^vs. completed program of intervention group.

Source: Author’s development.

In the intervention group, most participants performed aerobic training on a treadmill, while two participants utilized a stationary bike. Although not specified in the original study protocol,^20^ two children were transitioned to a circuit-based exercise format (including ball games, running, and trampoline jumping) to ensure better adherence. During aerobic training in the intervention group, the target HR was maintained between 65 and 85% of the peak HR achieved during the MST.

Nine volunteers in the control group and eight in the intervention group did not finish the protocol. Thus, the per-protocol analysis was performed. In the control group, no significant differences were observed in any baseline characteristics between participants who completed the study and those who did not. Similarly, in the intervention group, no baseline characteristics differed between groups, except for inhaled corticosteroid doses, which were higher in the non-completers (Table 1).

There was no difference in exercise capacity, distance walked, number of steps/day (sedentary behavior) (Table 2), quality of life (PAQLq total score), and inflammatory profile (Table 3) between the control group and intervention group.

**Table 2:** Exercise capacity and physical activity in daily living of children and adolescents receiving or not an 8-week exercise training program, on per-protocol analysis.

	Intervention (n=30)	Control (n=29)	Between-group change	p-value
VO_2_peak, ml/kg				
Baseline	40.0±12.7	38.3±12.5	1.6 (-5.0; 8.3)	0.626
Post-intervention	40.3±12.4	39.2±16.0	1.1 (-6.5; 8.7)	0.764
Within-group change	-0.3 (-5.5; 4.8)	-0.8 (-5.9; 4.2)		
p-value	0.893	0.741		
Peak workload, watts				
Baseline	91.4±45.4	80.6±36.0	10.8 (-10.8; 32.4)	0.320
Post-intervention	100.4±45.3	83.0±34.5	17.3 (-3.8; 38.5)	0.102
Within-group change	-9.0 (-13.7; 4.4)*	-2.5 (-7.1; 2.1)		
p-value	<0.001	0.284		
Modified shuttle walk test, m				
Baseline	817.5±181.2	761.4±207.0	56.0 (-46.3; 158.4)	0.278
Post-intervention	852.0±218.6	759.2±254.6	92.8 (-32.1; 217.6)	0.142
Within-group change	-34.5 (-85.6; 16.5)	2.2 (-48.9; 53.3)		
p-value	0.181	0.932		
Modified shuttle walk test, % pred				
Baseline	85.9±14.6	78.9±15.9	7.0 (-1.0; 15.0)	0.085
Post-intervention	90.6±18.6	82.4±15.2	8.1 (-0.7; 7.0)	0.072
Within-group change	-4.6 (-8.3; -1.0)*	-3.5 (-7.2; 0.2)		
p-value	0.013	0.065		
Slow/light physical activity, %				
Baseline	95.1±3.2	96.0±2.8	-0.8 (-2.5; 0.8)	0.318
Post-intervention	83.9±20.7	81.0±21.4	2.9 (-8.7; 14.5)	0.615
Within-group change	11.2 (3.1; 19.3)*	15.0 (6.7; 23.2)*		
p-value	0.008	0.001		
Number steps/day, total				
Baseline	8,358±2,366	7,834±3,280	523 (-1038; 2085)	0.504
Post-intervention	7,408±4,021	6,574±3,231	833 (-1158; 2825)	0.405
Within-group change	950 (-687; 2,588)	1,259 (-378; 2,897)		
p-value	0.250	0.129		

VO_2:_ peak oxygen uptake.

*Statistically significant differences (p<0.05).

Source: Author’s development.

**Table 3:** Quality of life and interleukin profile, on per-protocol analysis.

	Intervention (n=30)	Control (n=29)	Between-group change	p-value
PAQLq, total score				
Baseline	5.4±1.0	5.3±0.8	0.1 (-0.4; 0.6)	0.769
Post-intervention	5.9±0.6	6.5±3.4	-0.6 (-1.9; 0.6)	0.317
Within-group change	-0.5 (-1.3; 0.4)	-1.2 (-2.2; -0.2)		
p-value	0.310	0.015*		
	**Intervention** **(n=26)**	**Control** **(n=23)**		
IL–10, pg/mL				
Baseline	11.2±2.9	14.9±3.1	-3.6 (-12.3; 5.0)	0.401
Post-intervention	13.8±5.5	22.2±5.8	-8.5 (-24.6; 7.7)	0.023*
Within-group change	13.8 (2.7; 24.9)	22.2 (110.5; 34.0)		
p-value	0.401	0.298		
IL–13, pg/mL				
Baseline	119.4±61.0	109.2±64.9	10.2 (-169.1; 189.5)	0.909
Post-intervention	131.7±66.3	125.3±70.5	6.3 (-188.3; 201.1)	0.948
Within-group change	-12.3 (-38.6; 14.1)	-16.1 (-44.1; 11.9)		
p-value	0.355	0.254		
IL–17, pg/mL				
Baseline	7.8±2.9	11.1±3.0	-3.2 (-11.6; 5.1)	0.442
Post-intervention	10.8±5.4	17.0±5.7	-6.2 (-22.0; 9.6)	0.436
Within-group change	3.0 (-13.8; 7.9)	-5.9 (-17.5; 5.6)		
p-value	0.586	0.309		
IL–4, pg/mL				
Baseline	30.1±10.4	33.8±11.1	-3.7 (-34.4; 27.0)	0.809
Post-intervention	33.6±9.6	31.6±10.2	2.0 (-26.2; 30.3)	0.883
Within-group change	-3.5 (-15.8; 8.7)	2.2 (-10.8; 15.2)		
p-value	0.564	0.730		
IL–5, pg/mL				
Baseline	12.9±5.9	12.8±6.3	0.1 (-17.3; 17.5)	0.990
Post-intervention	15.5±1.8	18.5±8.5	-3.0 (-26.4; 20.3)	0.795
Within-group change	-2.6 (-8.9; 3.7)	-5.7 (-12.4; 0.951)		
p-value	0.411	0.091		
TNF-alfa, pg/mL				
Baseline	12.4±1.4	12.7±1.5	-0.3 (-4.5; 3.8)	0.869
Post-intervention	14.2±1.9	13.9±1.0	0.3 (-5.2; 5.8)	0.912
Within-group change	-1.8 (-5.0; 1.3)	-1.2 (-4.6; 2.2)		
p-value	0.261	0.495		

PAQLq: Pediatric Asthma Quality of Life Questionnaire; IL: interleukin; TNF-alfa: tumor necrosis factor.

*Statistically significant differences (p<0.05).

Source: Author’s development.

At baseline, 38% (n=11) of the control group and 57% (n=17) of the intervention group achieved normal functional capacity (≥80% at MST, p=0.251). Following the intervention, these proportions increased to 52% (n=15) and 73% (n=22), respectively (p<0.001, χ^2^ test). Regarding the absolute distance walked, 11 participants from the control group and 13 from the intervention group reached the 47-m threshold (p=0.673, χ^2^ test).

Regarding the inflammatory profile, no differences were observed in the inflammatory profile, except for IL-10. Although statistically significant, the observed difference in IL-10 levels between groups was of modest magnitude (Table 3).

## DISCUSSION

This is the first clinical trial to comprehensively assess the effects of an exercise program on exercise capacity, based on CPET and functional capacity, quality of life, sedentary behavior, and the inflammatory profile in children and adolescents with asthma.

After eight weeks of the exercise program, no significant differences were observed between the intervention and control groups regarding these outcomes.

Exercise training programs are consistently associated with moderate-to-large improvements in exercise capacity and quality of life among pediatric asthma patients.^5,6,7,8,9,10,11^ Although our sample characteristics and exercise protocol were similar to those in previous trials, we did not observe the same statistically significant improvements in exercise capacity previously reported. Regarding the protocol, three factors may explain why our results differ from those of other studies. First, the twice-weekly exercise frequency was chosen based on previous literature and to mirror “real-life” conditions; for many families, attending supervised sessions more frequently is not feasible due to time and logistical constraints. This pragmatic approach is consistent with recent study designs.^27^ Second, the control group underwent a supervised protocol, unlike other studies that utilized usual care or simple orientation; this may have led to an overestimation of their outcomes. Third, the sample comprised clinically stable participants, as all individuals had been on regular pharmacological treatment for six months, with no medication changes in the last three months.

Even though there were no statistical differences, clinically important changes were observed. In the CPET, a significant increase in workload was noted between groups following the exercise program. This difference exceeds the minimal clinically important difference,^22^ suggesting an improved aerobic capacity in the intervention group. Regarding functional capacity as assessed by the MST, a higher proportion of participants in the intervention group improved following the protocol compared with the control group. This finding suggests a positive, albeit modest, impact of the exercise program on functional performance.

No significant changes were observed in sedentary behavior, as measured by daily step counts. It is well-established that a sedentary lifestyle (8,000 steps/day) is closely related to, yet distinct from, low exercise capacity.^28^ Furthermore, physical inactivity and sedentary behavior are not synonymous.^28,29^ While the former refers to a failure to achieve the recommended 60 minutes of daily moderate-to-vigorous physical activity, sedentary behavior specifically describes time spent in seated or lying postures.^29^ Consequently, participants could potentially meet the 60-minute daily activity threshold while remaining sedentary for the remainder of the day. This duality may explain why we observed an increase in the number of volunteers reaching normal values in the MST (>80% predicted) despite no concurrent reduction in sedentary behavior.

Given these findings, a critical question remains: are current exercise protocols for children and adolescents with asthma truly consistent enough to foster lasting change? We suggest that to significantly improve exercise capacity and counteract sedentary habits, exercise programs should ideally be performed daily, rather than only twice a week. These findings reinforce the importance of adherence to the World Health Organization (WHO) guidelines,^29^ which recommend a higher frequency of physical activity (daily or at least several times a week) to achieve significant physiological benefits in children and adolescents with asthma on regular pharmacological treatment, regardless of disease severity. Additionally, these findings should be interpreted as evidence that a twice-weekly, 8-week supervised program was insufficient to produce statistically significant between-group improvements, rather than as definitive evidence that exercise training is ineffective in this population.

Our study has some limitations:

a) neither the participant nor the physical therapist who conducted the protocol was blinded because exercise protocols require the participant’s physical effort and awareness, and physiotherapists must know which exercise they are administering;
b) although we experienced a 20% dropout rate, a per-protocol analysis was maintained. Most dropouts were due to parents’ logistical constraints during workdays. Additionally, severe asthma patients were underrepresented, reflecting their lower prevalence;
c) we lack data regarding the number of volunteers in either group who may have engaged in physical activity more than twice a week outside the study protocol; and
d) the inflammatory profile could not be assessed for 15 volunteers (n=9 in the control group and n=6 in the intervention group) because the blood sample was not properly frozen.

In conclusion, a twice-weekly exercise program (16 sessions) was insufficient to improve exercise capacity, sedentary behavior, or quality of life in children and adolescents with asthma receiving regular pharmacological treatment. Therefore, different weekly exercise frequencies should be investigated in patients with asthma undergoing regular treatment.

## Data Availability

The database that originated the article is available with the corresponding author.
